# Is the 10 metre walk test on sloped surfaces associated with age and physical activity in healthy adults?

**DOI:** 10.1186/s11556-019-0219-0

**Published:** 2019-07-18

**Authors:** Daniel Thomson, Matthew Liston, Amitabh Gupta

**Affiliations:** 1 School of Science and Health, Western Sydney University, Sydney, Australia; 2 Centre for Human and Applied Physiological Sciences, School of Biomedical Sciences, King’s College London, London, UK

**Keywords:** Gait, Speed, Walking, Ageing, Inclined

## Abstract

**Background:**

Preferred walking speed is considered an important indicator of health in older adults and is measured on level ground. However, this may not represent the complex demands of community ambulation such as walking on sloped surfaces. Performing a 10 m walk test on a sloped surface is a novel test, and may be a more sensitive measure of walking capacity which may better discriminate age or health-related changes in gait speed compared to a traditional level 10 m walk test. The purpose of this investigation was to determine healthy adults’ performance in the 10 m walk test across various inclines and speeds, and which version of the 10 m walk test would be best at discriminating age-related changes in walking speed. Further, this study aimed to determine whether measures of general health and physical activity are associated with the performance of each test.

**Methods:**

Healthy Adults (*n* = 181) aged 20–80 years completed the 10 m walk test on level, downhill and uphill surfaces (8° inclination) at fastest and preferred speeds. Descriptive statistics were calculated for walking speed for males and females across each decade of life. Repeated measures ANOVA was performed to discriminate age-related changes in gait speed by decade, for the 10 m walk test at each speed and slope. Multiple linear regression analyses were conducted to examine the association between waist to height ratio, resting heart rate, age and self-reported physical activity upon preferred and fastest walking speeds at each incline (level/downhill/uphill).

**Results:**

The 10 m walk test best discriminated age-related changes in gait speed when performed at fastest speeds on each slope, or at a preferred speed on an uphill slope. Waist to height ratio, age and the physical activity index were all significantly associated with fastest walking speeds over each incline and preferred uphill speed. Only waist to height ratio was associated with preferred walking speed on level and downhill surfaces.

**Conclusions:**

The 10 m walk test has the greatest ability to discriminate age- and health-related changes in gait speed when it is performed at a fastest speed on any slope, or uphill at a preferred speed. The normative data reported in this study may be used to compare the performance of the 10 m walk test to that of healthy adults at preferred and fastest speeds on sloped surfaces.

**Electronic supplementary material:**

The online version of this article (10.1186/s11556-019-0219-0) contains supplementary material, which is available to authorized users.

## Introduction

Walking speed, often referred to as the ‘sixth vital sign’ is associated with an individual’s health [1] and the ease with which they can navigate the environment [2]. Walking speeds of less than 1 m.s^− 1^ are associated with negative health outcomes and are predictive of morbidity and mortality in the elderly [1, 2]. The 10 m walk test (10MWT) is a validated and reliable tool for determining walking speed in adults and can assess both preferred and fastest speeds [3]. The 10MWT has previously been used to describe differences in walking speed across the lifespan [3, 4], demonstrating that speed begins to decline at 50 years of age and continues to decrease in each progressive decade of life [5, 6]. These age-related changes in walking speed may be mediated by the amount of physical activity an individual performs, as less physically active older adults have been shown to walk slower than their age-matched counterparts [7]. The amount of physical activity an older adult performs is also related to the maintenance of independence in community ambulation, with less active adults having reduced functional capacities [8, 9]. Older adults who spend more time outside of their home are more physically active, walk for longer than those who spend more time indoors [8], and have significantly reduced risk of mortality if they walk for more than 1 hour daily [10]. However, walking tests such as the 10MWT which are currently utilised in clinical practice do not reflect the demands of community ambulation.

Walking in the community is complex, as individuals are required to traverse uneven or sloped surfaces, walk in crowded environments and perform concomitant tasks such as talking [11]. This increase in complexity may challenge older adults due to age-associated and deleterious changes in their cognitive, sensory and musculoskeletal systems [12–14]. The complexities of ambulating in the community may not be captured by the 10MWT, which is often performed indoors on level ground in enclosed spaces [4, 15]. Measurements of walking speed across different slopes may provide further information regarding how individuals manage challenging situations that are commonly experienced during community ambulation. Walking uphill has been shown to require greater power generation [16] whilst walking downhill has been shown to challenge balance in individuals more than level walking [17]. Sloped walking may be more difficult for individuals, particularly older adults, due to the relatively greater decline in muscle strength, balance and motor control [9]. Accordingly, a measure of walking speed on a sloped surface may be more sensitive to detect change across the lifespan and provide a more relevant description of how people modify their gait to task demands, particularly in community ambulant middle-aged and older adults. To date, no tests of walking speed on sloped surfaces have been reported, even though they are omnipresent in community settings.

It is plausible that walking speeds on sloped surfaces are associated with an individual’s physical activity, health and fitness. Physical activity, cognitive impairment and muscle weakness have all been associated with the development of slower walking speeds on level ground [18]. Since participation in physical activity is associated with the ability to walk outdoors and maintain general health [8, 10], physical activity, health and fitness may predict performance when walking on a sloped surface. The waist-to-height ratio (WHtR) is a measure of general health risk [19] and is reported to be predictive of morbidity, mortality, and disability in older adults [20] and resting heart rate (RHR) has been shown to predict fitness and the risk of mortality [21]. Self-reported measures of physical activity, such as the Global Physical Activity Questionnaire (GPAQ) and Physical Activity Index (PAI) have also been shown to correlate with the amount of physical activity performed in a week and provide a quick measure of someone’s physical activity behaviours [22, 23]. However, it is not known whether there is an association between walking speed on an incline and measures of health, fitness and self-reported physical activity.

The purpose of this investigation was to determine healthy adults’ performance in the 10MWT across various inclines and speeds. The primary aim was to provide normative data on fastest and preferred walking speeds when performed on sloped surfaces, for males and females across the lifespan. The secondary aims of the study were to (i) investigate which version of the 10MWT would be best at discriminating age-related changes in gait speed in healthy adults, and (ii) to determine the association between walking speed on sloped surfaces with age, self-reported physical activity and measures of health and fitness.

## Methods

### Participants

Healthy adults (*n* = 181) aged between 20 and 80 years were recruited (Table 1) by advertising posters and the use of online noticeboards in local community health centres and volunteer organisations in the local area. The determination of sample size in the current study was based upon previous studies which have provided normative data using similar tests of gait speed [3, 24, 25]. Further, it exceeded an estimated sample size of 82 participants required to test the secondary hypothesis, which involved a conservative estimate of 50 participants, plus a further 8 participants for each predictor variable (Age, GPAQ/PAI, RHR, WHtR) included in the multivariate regression analysis [26]. Purposive sampling ensured that there were an equal number of males and females included for each decade across the lifespan. Participants were excluded if they self-reported any known neuromuscular, musculoskeletal or cardiorespiratory disease; any impairment which may impact upon their current ability to walk in the community; or a report of a fall in the 12 months prior to testing. Ethical approval for the study was granted by the institutional Human Research Ethics Committee (H11410). Each participant provided written and informed consent prior to enrolment in the study.

**Table 1:** Participant characteristics (mean (SD)) for males and females across each decade of life

Group	Age	WHtR	RHR	PAI	GPAQ
20–29 (15:15)	22 (2)	0.44 (0.05)	70 (9)	6.69 (3.41)	2601 (1871)
30–39 (15:15)	34 (3)	0.48 (0.05)	73 (14)	4.44 (4.20)	2354 (2464)
40–49 (15:15)	44 (2)	0.53 (0.07)	71 (9)	5.08 (4.19)	2636 (2300)
50–59 (15:15)	55 (2)	0.52 (0.05)	72 (8)	3.10 (3.46)	2558 (3579)
60–69 (15:16)	63 (2)	0.53 (0.09)	65 (9)	3.90 (2.90)	1599 (1907)
70–80 (15:15)	73 (2)	0.56 (0.08)	69 (11)	3.10 (3)	1769 (2602)

Measures are provided for each group (age range in years (ratio of males to females)) across the lifespan including the waist-to-height ratio (WHtR), resting heart rate (RHR) (beats.min^− 1^) and scores for the Physical Activity Index (PAI) and Global Physical Activity Questionnaire (GPAQ) (Metabolic Equivalent minutes)

### Testing procedure

Each participant’s age, height, waist circumference (WHO, 2008) and RHR (Palatini et al., 2006) were measured and recorded. Each participant was then instructed to complete a series of 10MWT on level ground, uphill and downhill on a ramp of 8 degrees inclination (Digital Inclinometer, Baseline Evaluation Instruments, New York). Testing was conducted during dry, daytime conditions, on an asphalted surface with overhead cover, in a public place and in the absence of pedestrians. A distance of 14 metres was measured (Bosch GLM 40 Professional, Bosch, Germany) both on level ground and the sloped ramp, consisting of a 10 m length, and a 2 m section at either end for acceleration and deceleration to ensure steady state walking was achieved over the 10 m length [27]. Visible markers were placed on the ground at distances of 0, 2, 12 and 14 m to mark out the walking track. During testing the primary investigator stood adjacent to the walking track at approximately half the walking distance to measure the duration for the participant to walk 10 m (iOS Clock, Apple, California), and count the number of steps taken to complete the 10 m distance. When an individual did not step directly on the finish line, half steps were reported. During all walking trials participants were shod and wore loose comfortable clothes.

Each participant completed the 10MWT on each of the three surfaces (level, downhill, uphill) at their preferred walking speed and their fastest walking speed. Participants were instructed to “walk at your usual comfortable speed” and to “walk as fast as you safely can without running” for the preferred and fastest conditions, respectively [3]. Participants completed two trials at each speed on each surface. The first trial was used to familiarise participants to each condition and the second trial was always used for analysis. Participants always completed the trials at their preferred speed prior to the trials at their fastest speed. The level walking condition was always performed first for both speeds, after which participants completed walking on the sloped surfaces in a randomised order.

To estimate weekly physical activity, each participant completed the GPAQ, a validated self-completed 16 item questionnaire detailing average weekly physical activity [22]. Self-reported physical activity from the GPAQ was converted into Metabolic Equivalent (MET) minutes [28]. Subsequently, each participant completed the PAI which yields a score between 0 and 15 and describes the weekly recreational level of physical activity [23]. The RHR was measured at the radial pulse over a duration of 30 s [21] and the WHtR calculated (quotient of the waist circumference (m) to height (m)) [20]. Dependant variables including gait speed (m.s^− 1^), step length (m) and cadence (steps.min^− 1^) were calculated for the second trial of each of the six 10MWT conditions.

### Statistical analyses

Descriptive statistics for walking speed, step length and cadence were calculated for the sample for males and females categorised by decade of life (Microsoft Excel 2016, Microsoft). To examine the secondary research aim (i), a mixed repeated measures ANOVA was performed with 2 within subject factors (incline and speed) and 2 between subject factors (sex and decade of age). Normality was checked for variables prior to performing an ANOVA and regression analyses by visual inspection of Q-Q plots of standardized residuals. To compare the discriminative ability of each 10MWT condition across age groups, a priori between-group comparisons were made to compare the reference group of 20–29 year old adults to all other decades for gait speed, step length and cadence. Between-group a priori comparisons between males and females were also made for each decade. Normality for variables within each group was assessed using a Shapiro Wilk test, with significance accepted at *p* > 0.05. If the distribution was normal, an independent samples t-test was performed, and if not normally distributed a Mann Whitney U test was performed, with statistical significance accepted at *p* < 0.05 and effect sizes reported (partial eta squared (n^2^)). To examine the secondary research aim ii), multiple linear regression analyses were performed. A Shapiro-Wilk test of normality was performed for dependant variables including the GPAQ and PAI. A Pearson’s Correlation or Spearman’s Rho (ρ) was calculated depending upon normality to determine the correlation between the GPAQ and PAI. To determine whether the GPAQ or PAI was a stronger predictor of walking speed (preferred/fastest) on each incline (level/downhill/uphill), separate multiple linear regression analyses were performed. All analyses included input variables of age, WHtR and RHR which were combined with either the PAI or GPAQ in the linear regression models. The results from each of these multiple linear regressions (PAI, GPAQ) were compared using the adjusted R^2^ value to determine the model with the best fit, along with the contribution of each self-reported physical activity measure compared using the partial eta squared (n^2^) for a measure of effect size. Minimum to maximum ranges for model fit (R^2^) and effect size (n^2^) were provided for the six 10MWT conditions for the GPAQ and PAI. The self-reported physical activity measure which demonstrated the best model fit was then used to answer the secondary aim of the study. The overall model fit for each 10MWT was expressed as the adjusted R^2^ value, with statistical significance for each input variable being accepted at *p* < 0.05 and effect sizes for each input variable were expressed as n^2^ values. All statistical tests were performed in IBM SPSS Statistics Version 24 (IBM, New York).

## Results

**Fig. 1:**
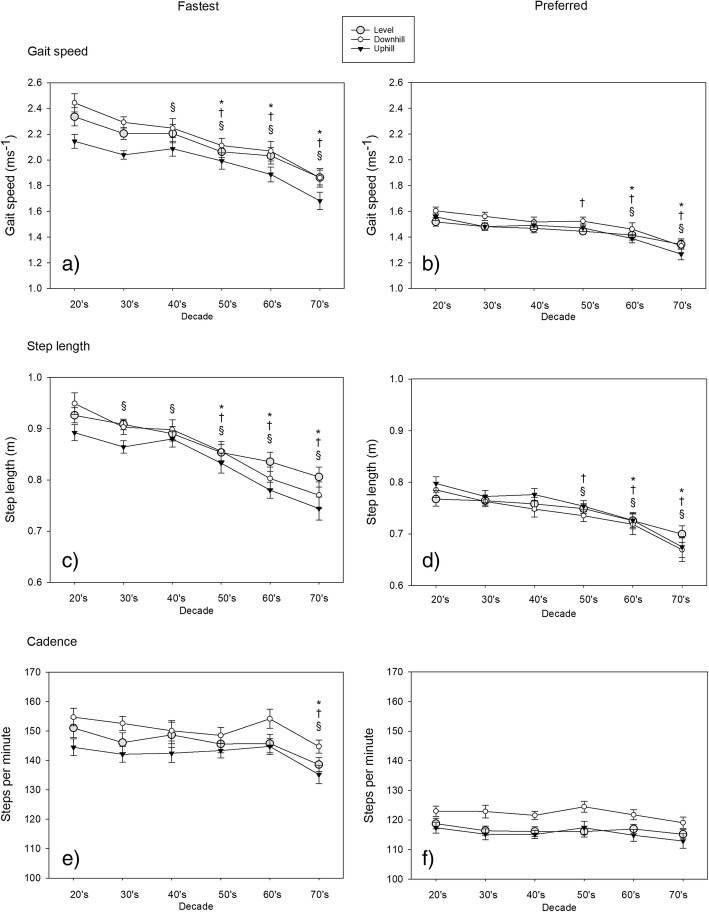
Mean (SE) data for gait speed, step length and cadence sorted by decade of life. Mean ± SE gait speed (m.s^− 1^), step length (m) and cadence (steps per minute), are provided for fastest (a, b, c) and preferred (d, e, f) trials respectively for each decade. The level 10MWT is shown as the grey shaded circle, downhill 10MWT is the white circle, and uphill is the shaded triangle. * *p* < 0.05 for level condition compared with 20–29 year old adults § *p* < 0.05 for downhill condition compared with 20–29 year old adults † *p* < 0.05 for uphill condition compared with 20–29 year old adults

A total of 181 participants completed all 10MWT. Descriptive statistics of gait speed, step length and cadence were categorised by decade for each sex (Table 2) (Fig. 1).

**Table 2:** Model fit (adjusted R^2^) and effect size (n^2^) for the Physical Activity Index (PAI) and Global Physical Activity Questionnaire (GPAQ) for each of the 10MWT conditions

	PAI		GPAQ	
	n^2^	Adjusted R^2^	n^2^	Adjusted R^2^
Preferred Level	0.01	0.182	0.005	0.178
Preferred Downhill	0.019	0.231	0.003	0.219
Preferred Uphill	0.03	0.258	0.011	0.243
Fast Level	0.026	0.261	0.003	0.243
Fast Downhill	0.029	0.306	0.014	0.295
Fast Uphill	0.062	0.256	0.016	0.22

There were significant effects of instructions to walk at a preferred or fastest speed (*F*_*(1,169)*_ = 1149.93, *p* < 0.01, n^2^ = .87), the slope of walking (*F*_*(2,338)*_ = 87.40, *p* < 0.01, n^2^ = .34), sex (*F*_*(1,169)*_ = 17.07, *p* < 0.01, n^2^ = .09) and decade of age (*F*_*(5,169)*_ = 11.69, *p* < 0.01, n^2^ = .26) for walking speed across all testing conditions. There were also significant effects of instructions to walk at the preferred or fastest walking speed (*F*_*(1,169)*_ = 854.65, *p* < 0.01, n^2^ = .84), sex (*F*_*(1,169)*_ = 71.48, *p* < 0.01, n^2^ = .30), decade of age (*F*_*(5,169)*_ = 15.15, *p* < 0.01, n^2^ = .31) and slope of walking (*F*_*(2,338)*_ = 9.82, *p* < 0.01, n^2^ = .06) on step length for all testing conditions. Only the factors of the instruction to walk at a preferred or fast speed (*F*_*(1,169)*_ = 698.50, *p* < 0.01, n^2^ = .81), the slope of walking (*F*_*(2,338)*_ = 90.20, *p* < 0.01, n^2^ = .35) and sex (*F*_*(1,169)*_ = 10.20, *p* = 0.02, n^2^ = .06) had a significant effect on walking cadence.

Walking speed was significantly slower for the fastest, downhill condition only, for 40–49 year olds relative to 20–29 year olds (*p* = 0.02, Z = 2.43, n^2^ = .10). Adults aged 50–59 walked significantly more slowly than 20–29 year old adults during their fastest effort on the level, downhill and uphill walking slopes (fast level and uphill; *p* ≤ 0.03, Z ≥ 2.24, n^2^ ≥ .08, fast downhill; *p* < 0.01, t = 3.71, n^2^ = .19), as well as during preferred effort on an uphill slope (*p* = 0.05, t = 2.05, n^2^ = .07). Adults aged 60–69 and 70–79 years walked significantly more slowly than 20–29 year old adults during all 10MWT conditions (60–69 year old, fast level - *p* < 0.01, Z = 3.04, n^2^ = .15; preferred level - *p* < 0.05, t = 2.04, n^2^ = .06; fast/preferred downhill and uphill - *p* ≤ 0.01, t ≥ 3.20, n^2^ ≥ .14; 70–79 year old: fast level - *p* < 0.01, Z = 4.66, n^2^ = .36; preferred level - *p* < 0.01, t = 3.67, n^2^ = .18; fast/preferred downhill and uphill - *p* < 0.01, t ≥ 4.42, n^2^ ≥ .25).

Adults in their 30’s and 40’s took significantly shorter steps than adults in their 20’s during the fastest effort, downhill (30’s - *p* = 0.02, Z = 2.40, n^2^ = .10; 40’s - *p* = 0.03, Z = 2.15, n^2^ = .08). Adults in their 50’s took significantly shorter steps than adults in their 20’s during all variations of the 10MWT other than preferred level walking (fast downhill/uphill and preferred uphill - *p* ≤ 0.02, Z ≥ 2.38, n^2^ ≥ .09; fast level/preferred downhill - *p* ≤ 0.01, t ≥ 2.68, n^2^ ≥ .11). Adults in their 60’s and 70’s took shorter steps than young adults in all 10MWT conditions (60’s: preferred uphill - *p* < 0.01, t = 3.85, n^2^ = .20; all other 10MWT conditions *p* ≤ 0.03, Z ≥ 2.22, n^2^ ≥ .08) (70s: fast uphill/downhill and preferred downhill - *p* < 0.01, Z ≥ 4.00, n^2^ ≥ .27; fast level and preferred level/uphill - *p* < 0.01, t ≥ 4.88, n^2^ ≥ .28). Adults in their 70’s also had a significantly lower cadence during all fast 10MWT conditions compared with 20–29 year old adults (*p* ≤ 0.03, t ≥ 2.19, n^2^ ≥ .07).

**Fig. 2:**
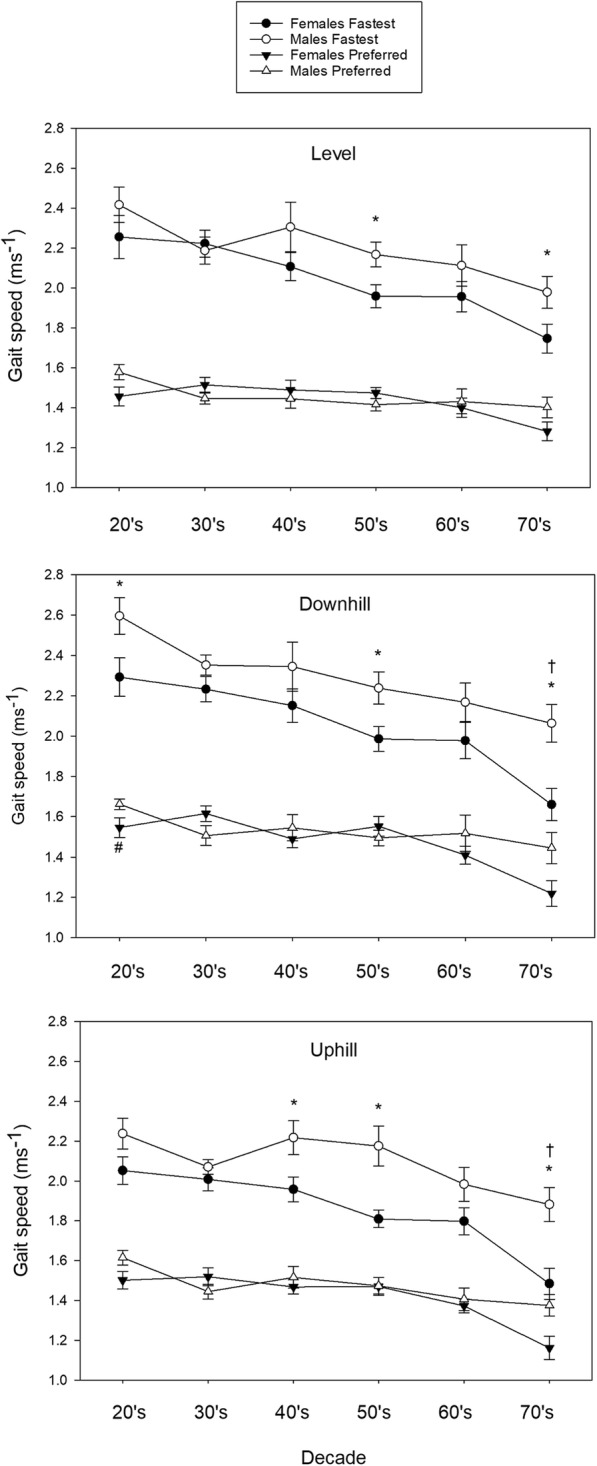
Walking velocity (m.s^− 1^) (mean (SE)) for males and females for each decade across the lifespan. Mean ± SE gait speed (m.s^− 1^) is provided for fastest and preferred level, downhill and uphill 10MWT trials separated by sex. * *p* < 0.05 for males compared with females for fastest speeds in the same decade. † *p* < 0.05 for males compared with females for preferred speeds in the same decade

Significant differences between males and females within each decade for walking speed are displayed in Fig. 2. Between-sex differences in step length and cadence are summarised in Additional file 1.

There was a statistically significant correlation (Spearman’s Rho) between the PAI and GPAQ (*r*^s^ = .591, *p* = < 0.001). Multiple regression analyses performed separately using the PAI and GPAQ demonstrated that the PAI yielded slightly higher adjusted R^2^ values, and partial eta squared for each version of the 10MWT when compared with the GPAQ (Table 2). As a result, the PAI was the self-reported physical activity measure that was chosen for analysis to answer the secondary aim.

Separate multiple regression analyses were used to predict each walking speed from age, WHtR, RHR and PAI. All walking speeds were significantly predicted by the model (*p* < 0.01) (Table 3). During preferred walking speeds, WHtR was a significant predictor for trials on level and downhill slopes, whilst WHtR, age and PAI were all significant predictors of preferred walking speed on an uphill surface respectively. Fastest level and downhill walking speeds were significantly associated with WHtR, age and PAI respectively, whereas fastest uphill walking speed was significantly associated with PAI, age and WHtR (Table 3). The RHR was not significantly associated with walking speed on any incline or speed.

**Table 3:** Predictor variables (*p* < 0.05) for preferred and fastest walking speeds on each slope (level, downhill and uphill)

	Fast level (*F* = 16.91, *R*^*2*^ = .261)	Fast downhill (*F* = 20.83, *R*^*2*^ = .306)	Fast uphill (*F* = 16.47, *R*^*2*^ = .256)	Preferred level (*F* = 11.01, *R*^*2*^ = .182)	Preferred downhill (*F* = 14.51, *R*^*2*^ = .231)	Preferred uphill (*F* = 16.66, *R*^*2*^ = .258)
Age	*p = .001, n^2^ = .063	*p ≤ .001, n^2^ = .081	*p = .001, n^2^ = .057	*p* = .082, n^2^ = .017	*p* = .096, n^2^ = .016	*p = .002, n^2^ = .052
WHtR	*p = .001, n^2^ = .067	*p ≤ .001, n^2^ = .079	*p = .006, n^2^ = .042	*p ≤ .001, n^2^ = .086	*p ≤ .001, n^2^ = .120	*p ≤ .001, n^2^ = .080
RHR	*p* = .252, n^2^ = .007	*p* = .189, n^2^ = .010	*p* = .460, n^2^ = .003	*p* = .547, n^2^ = .002	*p* = .755, n^2^ = .001	*p* = .748, n^2^ = .001
PAI	*p = .030, n^2^ = .026	*p = .023, n^2^ = .029	*p = .001, n^2^ = .062	*p* = .187, n^2^ = .010	*p* = .068, n^2^ = .019	*p = .021, n^2^ = .030

Overall model fit (adjusted R^2^) and effect size (n^2^) are provided for each model and variable respectively

## Discussion

The aims of the current study were to determine whether variations of the 10MWT performed at preferred or fastest speeds on various slopes would better discriminate changes in age, health risk or physical activity behaviours in healthy adults. The main findings of this study were that the 10MWT performed at fastest speeds on downhill, uphill and level slopes, and at preferred speeds on an uphill slope were best able to discriminate age-related changes in walking speed, and that walking speed was most influenced by waist to height ratio, age and self-reported physical activity when walking was measured at a participants’ fastest speed on sloped surfaces. The WHtR was most strongly associated with fastest downhill walking speed, and PAI was most strongly associated with fastest uphill walking speed. Therefore, the 10MWT performed at fastest speeds on sloped surfaces may be a more useful tool than a preferred 10MWT on level ground in discriminating age and health related changes in clinical practice.

The normative data presented in this study may be used as a point of reference for outdoor walking on sloped surfaces. Preferred walking speed on level ground has previously been used to determine an older adult’s morbidity, risk of mortality [2], and performance in activities of daily living that are important in maintaining independence [29]. This study indicates that when the 10MWT is performed on a sloped surface at an individual’s fastest speed, that it provides a more sensitive measure to detect earlier age-related changes in healthy adults compared to the 10MWT on level ground. Fast downhill walking discriminated the performance of adults when aged 40–49 years onwards compared to the reference group of the youngest adults, whilst fast level, and fast and preferred uphill walking discriminated 50–59 year old adults from those aged 20–29 years. Differences in walking speed between males and females were also most pronounced during fast walking conditions. These results indicate that for healthy adults, the downhill 10MWT at a fastest speed was most sensitive to detecting age-related changes, and may be most suitable for community-dwelling, healthy adults.

Changes in 10MWT performance on sloped surfaces also appear to be related to the health risk and physical activity status of an individual. There were stronger associations between WHtR, age and PAI during fast downhill walking compared with fast level walking, and with age and PAI for preferred uphill walking compared with preferred level walking. Since age, general health status and physical activity are related to independence in accessing the community [8, 9], it is plausible that the downhill and uphill 10MWT’s may be more sensitive in measuring functional capacity than the level 10MWT. It needs to be determined whether relatively slower inclined walking speeds are associated with the avoidance of walking in the community, or an increased risk of falling.

The WHtR and PAI had the strongest associations with walking speed on a sloped surface. As a single measure, the WHtR was the strongest predictor of preferred and fastest walking speed in both the level and downhill conditions, as well as during preferred uphill walking, with lower WHtR associated with greater walking speed for all conditions. The WHtR has been used as a marker of cardiovascular health and general health [19, 30], similar to waist circumference which has been associated with slower walking speeds and disability in older adults [20]. The link between and increased risk of poorer health and walking speed extends to inclined walking, shown by the strong association between WHtR and downhill and uphill speeds. The PAI was the strongest predictor of fastest uphill walking speed, whereby a higher PAI was associated with faster uphill walking speed. The observation that WHtR and PAI were associated with walking speed may demonstrate that overall health risk and physical activity behaviours may be more important factors than age in the preservation of gait speed over various inclines, among healthy, community dwelling adults. Therefore, modifying an individual’s physical activity behaviours or health risk may lead to a greater capacity to walk in the community.

The difference in associations between WHtR and PAI and walking speeds on various inclines are likely affected by the difference in demand that each sloped surface presents. For example, uphill walking requires greater propulsive force from the ankle and hip to allow relatively greater vertical displacement and consequently, may require greater metabolic and mechanical work at the lower limb [31]. In contrast, walking downhill may require greater control of balance due to having to lower the body and avoid falling forwards [16, 32]. An increased PAI reflects greater participation in weekly physical activity, which may lead to greater strength or a reduction in the age-related decline in strength in lower limb muscles, thereby assisting power generation to increase uphill walking speed. This is consistent with findings that decreases in plantarflexor strength [33] and a reduction in ankle joint torques [34] have been observed in older adults and may limit their ability to walk quickly up an inclined surface. Slower uphill walking speeds (compared to age- and sex-matched peers), may be ameliorated by increased participation in physical activity, although this requires verification in future research studies. The WHtR had the strongest association with downhill walking speed and it is plausible that having a relatively greater body mass (higher WHtR) may lead to a diminished ability to absorb and control the relatively larger joint forces imposed at the knee during downhill walking [16]. A strategy of walking slowly downhill may act to decrease the relatively higher joint forces at the knee for those with a higher WtHR, or to reduce the perceived threat of stumbling or falling to protect the neuromusculoskeletal system.

The PAI was found to be a better predictor of walking speed than the GPAQ. Therefore, for estimating an adult’s gait speed the PAI may be an effective tool which is quick and easy to perform, as it only requires three multiple choice questions to be answered [23]. Both the PAI and GPAQ have been shown to moderately correlate to physical activity measured by accelerometers [22, 23]. It is possible that the additional sensitivity of the PAI in estimating exercise intensity, with three options provided for the question “How hard do you push yourself?”, may enhance the utility of the measure in predicting gait speed when compared with the GPAQ. The PAI has also previously been used to predict peak VO_2_ in healthy male adults [23], and it may better estimate vigorous activity when compared with the GPAQ. Performing regular vigorous physical activity may have a stronger correlation with the potential to preserve muscle strength and the ability to walk quickly [35].

Participants in the current study were healthy, and purposive sampling ensured an equal number of participants were recruited for each decade of life from 18 to 80 years of age. The PAI and GPAQ may not have captured a representative sample of people with a diversity in the level of physical activity which is likely to more accurately represent the broader community. People who live in the community, especially elderly and older adults, often have comorbidities such as arthritis, heart disease or other illness which can affect their exercise tolerance, balance, and strength, thereby affecting their walking speed [14, 29]. Although the current study demonstrated a strong association between the fastest walking speed and WHtR and PAI, it needs to be determined whether these relationships are also found in people who are community ambulant and have comorbidities. Other factors such as sensory processing, muscle strength and postural control have been associated with level walking speed for community dwelling older adults including those with comorbidities [14]. These factors may have a stronger association with walking speeds on sloped surfaces due to the greater mechanical and metabolic demands, and greater decline in capacity of the physiological systems with increasing age. It is plausible that the results of the current study would differ if the 10MWT were performed on a slope of a different angle, as incline-dependent neuromuscular and biomechanical changes have been shown during walking [16, 36]. An angle of 8 degrees as used in the current study, represented the greatest suggested slope allowable as specified by the building code in Australia [37], and therefore was likely to lead to one of the greatest demands encountered by community ambulant individuals.

## Conclusion

When the 10MWT was performed on a sloped surface at an individual’s fastest speed, it was more sensitive in detecting age-related changes in walking speed for healthy adults. An individual’s waist to height ratio, age and self-reported physical activity behaviours are predictive of their gait speed across different inclines, and may be predictive of an individual’s capacity for community ambulation. The values presented in the current study may serve as normative data to allow for the comparison of walking speed on sloped surfaces.

## Additional file

Additional file 1: Walking speed (m.s^-1^), step length (m) and cadence (steps.min^-1^) organised by males and females in each decade. * *p*<0.05 significant difference for males compared with females of the same decade. (DOCX 16 kb)

## Data Availability

The datasets used and/or analysed during the current study are available from the corresponding author on reasonable request.
