# Supplementary material for: Is the 10 metre walk test on sloped surfaces associated with age and physical activity in healthy adults?
Source: Eur Rev Aging Phys Act. 2019 Jul 18;16:11. doi: 10.1186/s11556-019-0219-0 (PMC6639969; doi:10.1186/s11556-019-0219-0)
Supplement: Supplementary file 1 — Walking speed (m.s-1), step length (m) and cadence (steps.min-1) organised by males and females in each decade. * p<0.05 significant difference for males compared with females of the same decade. (DOCX 16 kb) [file 11556_2019_219_MOESM1_ESM.docx]

| Walking speed (m.s^-1^) | | | | | | | |
| --- | --- | --- | --- | --- | --- | --- | --- |
| Age | Sex | Preferred Level | Preferred Downhill | Preferred Uphill | Fast Level | Fast Downhill | Fast Uphill |
| 20-29 | M | 1.58 (0.15) | 1.66 (0.10)* | 1.61 (0.14) | 2.42 (0.34) | 2.60 (0.35)* | 2.24 (0.30) |
|  | F | 1.46 (0.18) | 1.55 (0.19) | 1.50 (0.17) | 2.26 (0.42) | 2.29 (0.37) | 2.05 (0.27) |
| 30-39 | M | 1.45 (0.11) | 1.51 (0.19) | 1.44 (0.14) | 2.19 (0.26) | 2.35 (0.19) | 2.07 (0.14) |
|  | F | 1.52 (0.14) | 1.62 (0.15) | 1.52 (0.17) | 2.22 (0.26) | 2.23 (0.24) | 2.01 (0.22) |
| 40-49 | M | 1.45 (0.18) | 1.55 (0.25) | 1.52 (0.21) | 2.30 (0.48) | 2.34 (0.47) | 2.22 (0.33)* |
|  | F | 1.49 (0.19) | 1.49 (0.17) | 1.47 (0.14) | 2.11 (0.27) | 2.15 (0.32) | 1.96 (0.24) |
| 50-59 | M | 1.42 (0.12) | 1.50 (0.15) | 1.47 (0.16) | 2.17 (0.24)* | 2.24 (0.31)* | 2.18 (0.39)* |
|  | F | 1.47 (0.11) | 1.55 (0.19) | 1.47 (0.17) | 1.96 (0.22) | 1.99 (0.24) | 1.81 (0.17) |
| 60-69 | M | 1.43 (0.24) | 1.52 (0.35) | 1.41 (0.22) | 2.11 (0.40) | 2.17 (0.37) | 1.98 (0.33) |
|  | F | 1.40 (0.19) | 1.41 (0.18) | 1.37 (0.14) | 1.96 (0.30) | 1.98 (0.36) | 1.80 (0.27) |
| 70-80 | M | 1.40 (0.20) | 1.44 (0.30)* | 1.37 (0.21)* | 1.98 (0.31)* | 2.06 (0.36)* | 1.88 (0.33)* |
|  | F | 1.28 (0.18) | 1.22 (0.25) | 1.16 (0.23) | 1.75 (0.28) | 1.66 (0.31) | 1.48 (0.30) |

*p<0.05

| Step length (m) | | | | | | | |
| --- | --- | --- | --- | --- | --- | --- | --- |
| Age | Sex | Preferred Level | Preferred Downhill | Preferred Uphill | Fast Level | Fast Downhill | Fast Uphill |
| 20-29 | M | 0.79 (0.06) | 0.81 (0.04)* | 0.81 (0.06) | 0.96 (0.07)* | 1.01 (0.07)* | 0.93 (0.07)* |
|  | F | 0.74 (0.08) | 0.76 (0.11) | 0.78 (0.08) | 0.89 (0.08) | 0.89 (0.12) | 0.85 (0.08) |
| 30-39 | M | 0.77 (0.04) | 0.78 (0.05) | 0.78 (0.06) | 0.94 (0.04)* | 0.95 (0.06)* | 0.9 (0.06)* |
|  | F | 0.76 (0.05) | 0.75 (0.06) | 0.76 (0.07) | 0.87 (0.05) | 0.85 (0.06) | 0.82 (0.05) |
| 40-49 | M | 0.77 (0.07) | 0.77 (0.09) | 0.8 (0.07) | 0.93 (0.08)* | 0.94 (0.11)* | 0.92 (0.08)* |
|  | F | 0.74 (0.07) | 0.72 (0.07) | 0.76 (0.05) | 0.85 (0.06) | 0.86 (0.10) | 0.84 (0.09) |
| 50-59 | M | 0.77 (0.04)* | 0.75 (0.05) | 0.78 (0.06)* | 0.92 (0.06)* | 0.93 (0.07)* | 0.91 (0.08)* |
|  | F | 0.73 (0.05) | 0.72 (0.07) | 0.72 (0.05) | 0.79 (0.06) | 0.78 (0.07) | 0.75 (0.06) |
| 60-69 | M | 0.75 (0.10) | 0.75 (0.13) | 0.75 (0.09) | 0.88 (0.11)* | 0.85 (0.12)* | 0.82 (0.08)* |
|  | F | 0.7 (0.07) | 0.68 | 0.7 (0.05) | 0.79 (0.07) | 0.76 (0.10) | 0.74 (0.08) |
| 70-80 | M | 0.75 (0.07)* | 0.74 (0.10)* | 0.74 (0.09)* | 0.88 (0.08)* | 0.87 (0.11)* | 0.83 (0.08)* |
|  | F | 0.65 (0.08) | 0.6 (0.10) | 0.61 (0.09 | 0.73 (0.08) | 0.67 (0.10) | 0.66 (0.09) |

*p<0.05

| Cadence (steps.min^-1^) | | | | | | | |
| --- | --- | --- | --- | --- | --- | --- | --- |
| Age | Sex | Preferred Level | Preferred Downhill | Preferred Uphill | Fast Level | Fast Downhill | Fast Uphill |
| 20-29 | M | 119.80 (9.51) | 123.34 (9.44) | 119.46 (9.22) | 150.88 (16.79) | 154.50 (17.13) | 143.95 (17.03) |
|  | F | 117.73 (10.08) | 122.64 (9.89) | 115.35 (11.00) | 151.23 (19.90) | 154.94 (16.44) | 144.99 (14.51) |
| 30-39 | M | 112.38 (7.75)* | 116.20 (10.64)* | 110.83 (10.01)* | 139.50 (17.33)* | 148.24 (11.00 | 137.83 (12.13) |
|  | F | 120.41 (7.89) | 129.56 (9.00) | 119.59 (8.54) | 152.62 (17.65) | 156.92 (13.61) | 146.37 (16.48) |
| 40-49 | M | 112.15 (8.42)* | 119.75 (8.20) | 113.75 (8.85) | 149.27 (29.40) | 150.20 (24.40) | 145.35 (22.37) |
|  | F | 120.08 (8.05) | 123.35 (6.56) | 116.52 (6.41) | 148.10 (16.31) | 149.95 (11.28) | 139.58 (11.21) |
| 50-59 | M | 110.14 (7.70)* | 119.01 (8.30)* | 113.00 (10.29)* | 142.27 (15.15) | 143.58 (14.70) | 142.67 (16.44) |
|  | F | 122.04 (8.13) | 129.95 (9.27) | 121.80 (12.11) | 148.95 (17.96) | 153.46 (14.20) | 144.06 (11.39) |
| 60-69 | M | 114.72 (7.28) | 119.91 (11.41) | 112.28 (8.94) | 142.90 (17.67) | 151.80 (19.20) | 144.67 (16.51) |
|  | F | 119.04 (9.29) | 123.44 (6.79) | 117.27 (12.20) | 148.36 (16.67) | 156.39 (16.75) | 144.90 (13.01) |
| 70-80 | M | 111.95 (7.97)* | 115.86 (9.92) | 111.32 (8.30) | 134.50 (12.14) | 141.70 (12.83) | 135.36 (18.11) |
|  | F | 118.43 (8.43) | 122.30 (10.81) | 114.49 (17.53) | 142.63 (12.89) | 147.78 (11.30) | 135.04 (16.84) |

*p<0.05
